# Deep transfer learning strategy for efficient domain generalisation in machine fault diagnosis

**DOI:** 10.1038/s41598-023-33887-5

**Published:** 2023-04-24

**Authors:** Supriya Asutkar, Siddharth Tallur

**Affiliations:** 1 Centre for Research in Nanotechnology & Science (CRNTS), IIT Bombay, Powai, Mumbai, Maharashtra 400076 India; 2 Department of Electrical Engineering (EE), IIT Bombay, Powai, Mumbai, Maharashtra 400076 India

**Keywords:** Electrical and electronic engineering, Applied mathematics

## Abstract

Automated fault diagnosis algorithms based on vibration sensor recordings play an important role in determining the state of health of the machines. Data-driven approaches demand a large amount of labelled data to build reliable models. The performance of such lab-trained models degrades when deployed in practical use cases in the presence of distinct distribution target domain datasets. In this work, we present a novel deep transfer learning strategy that fine-tunes the trainable parameters of the lower (convolutional) layers with respect to the changing target domain datasets and transfers the parameters of the deeper (dense) layers from the source domain for efficient domain generalisation and fault classification. The performance of this strategy is evaluated by considering two different target domain datasets and studying the sensitivity of fine-tuning individual layers in the networks using time-frequency representations of the vibration signals (scalograms) as inputs. We observe that the proposed transfer learning strategy yields near-perfect accuracy, even for use cases where low-precision sensors are used for data collection and unlabelled run-to-failure data with a limited number of training samples.

## Introduction

Automated fault diagnosis in machines based on sensor data is an emerging area of interest in condition-based monitoring (CBM) and industrial internet of things (IIoT), that enables improved production efficiency and lower risk of accidents in complex mechanical systems^1^. Unlike monitoring parameters such as surface temperature, power consumption, and presence of wear particles in lubricants, vibration-based fault diagnosis is now a well-established method for identifying incipient faults^2^. Methods for fault diagnosis can be divided into two broad categories: physics-based and data-driven approaches. Physics-based models require establishing a relationship between fault signatures in sensor parameters and progression of damage in mechanical parts of the machinery and typically encompass designing models based on dynamics, finite element method, and modal analysis e.g. parametric model for planetary gears developed by Xue et al.^3^. Implementation of such methods requires an in-depth understanding of the dynamics of the machinery, and customisation for every sensor installation, to account for variations in the ambient environment, mounting, and manufacturing parameters. Several data-driven algorithms have been reported for machine health monitoring, ranging from simple classifiers such as support vector machines (SVM)^4–6^, advanced classifiers like least square interactive support matrix machine (LSISMM)^7^, artificial neural network (ANN)^8^ to complex approaches based on variations of deep convolutional neural networks (CNNs)^9–13^.

Although significant improvement in fault classification accuracy is possible using deep learning techniques, their performance is contingent on two key requirements: (a) data used for training (source domain) and testing (target domain) should belong to the same distribution, and (b) the necessity of a large labelled training dataset for higher accuracy. This is impractical in real-world scenarios since data distribution is sensitive to changes in ambient environment and installation, variation in power and load, etc. This change in data distribution from the source domain dataset used for training the algorithm to the target domain dataset used for testing is labelled as *domain shift*. To mitigate the problem of domain shift in deep learning models, various transfer learning (TL) strategies are employed wherein knowledge gained from the source domain is leveraged to improve classification ability in the distinctly distributed target domain datasets. Among the various approaches, feature-based TL has shown good domain adaptation capability by reducing the distribution difference between the source and the target domain dataset. Xiao et al. have proposed a domain adaptive motor fault diagnosis technique that uses CNN to extract multi-level features from the raw vibration data and maximum mean discrepancy (MMD) is adopted in the training process to reduce the distribution difference between the source and the target domain dataset. Thus, the knowledge learned from the source domain that possesses labelled motor vibration data under invariant working conditions is used to improve fault classification accuracy when the target domain belongs to the unlabelled data under constantly varying working conditions^14^. Zhao et al. have proposed an intelligent gearbox fault diagnosis method based on adaptive intraclass and interclass convolutional neural network (AIICNN) to improve generalisation under varying working conditions^15^. In another study, a deep convolution transfer learning network (DCTLN) is proposed that comprises a condition recognition module along with a domain adaptation module to effectively learn domain-invariant features of the target domain datasets. The DCTLN trained with labelled data of the source domain dataset is able to effectively classify unlabelled data of the target domain dataset^16^. Tong et al. have presented an unsupervised fault diagnosis approach incorporating feature TL to efficiently adapt to the varying working conditions of the target domain dataset^17^. Qian et al. on the other hand, have proposed an improved joint distribution adaptation (IJDA) technique to align not only the marginal but conditional distributions of the source and the target domain datasets for effective domain generalisation when validated using bearing and gearbox vibration signal datasets^18^. However, these techniques typically require training architectures from scratch and thus, can be computationally expensive.

Parameter-based TL on the other hand is a commonly employed strategy, wherein only a few layers of large pre-trained networks (e.g. VGG-16, ResNet-50, etc.) are fine-tuned while freezing a large number of the layers, for domain generalisation to a diverse set of target image datasets^19^. This approach is suitable for CBM applications that represent the vibration signals as encoded images through suitable time-frequency transforms e.g. spectrogram, scalogram, etc. Shao et al. have presented a novel deep TL framework comprising a pre-trained VGG-16 network that is trained on the ImageNet dataset which later is fine-tuned using the time-frequency images (scalograms) of the target machine vibration dataset. In the process, only the three highest-level blocks of the pre-trained VGG-16 network are fine-tuned while leaving the weights of the bottom blocks frozen to achieve state-of-the-art accuracy when validated against the three publicly available machine vibration data^20^. Wen et al. proposed a TL strategy that includes the use of a pre-trained ResNet-50 network to identify fault by fine-tuning just the fully connected layer added on top of the ResNet-50 network with respect to the publicly available machine vibration datasets^21^. Since these pre-trained networks are trained on the ImageNet dataset, consisting of images significantly different from the vibration encodings of the machines (spectrograms and scalograms), large networks are required for good adaptation ability. This results in a large number of trainable parameters and memory consumption along with additional post-processing on the target domain images. To address this challenge, recently, several reports for domain adaptation in vibration CBM using TL-CNNs operating on raw time-series vibration data have been reported, albeit with feature transfer and domain adversarial network or parameter transfer in dense layers of CNN^22–24^. However, in most TL reports for CBM, high-precision sensors or publicly available high-resolution datasets are used in the target domain, and domain generalisation of TL models for vastly different target domain data with low-precision sensors has not been adequately explored. CBM with low-cost consumer-grade sensors is essential for scaling the benefits of predictive maintenance to large factories, wherein instrumenting every machine with multiple high precision and expensive piezoelectric vibration sensors is impractical^25, 26^. Another challenge in the deployment of deep learning techniques pertains to the acquisition of faulty data sets in the field, as machines may stop working abruptly and therefore result in a disproportionate amount of healthy data being collected over data in faulty operating conditions^27, 28^.

**Figure 1:**
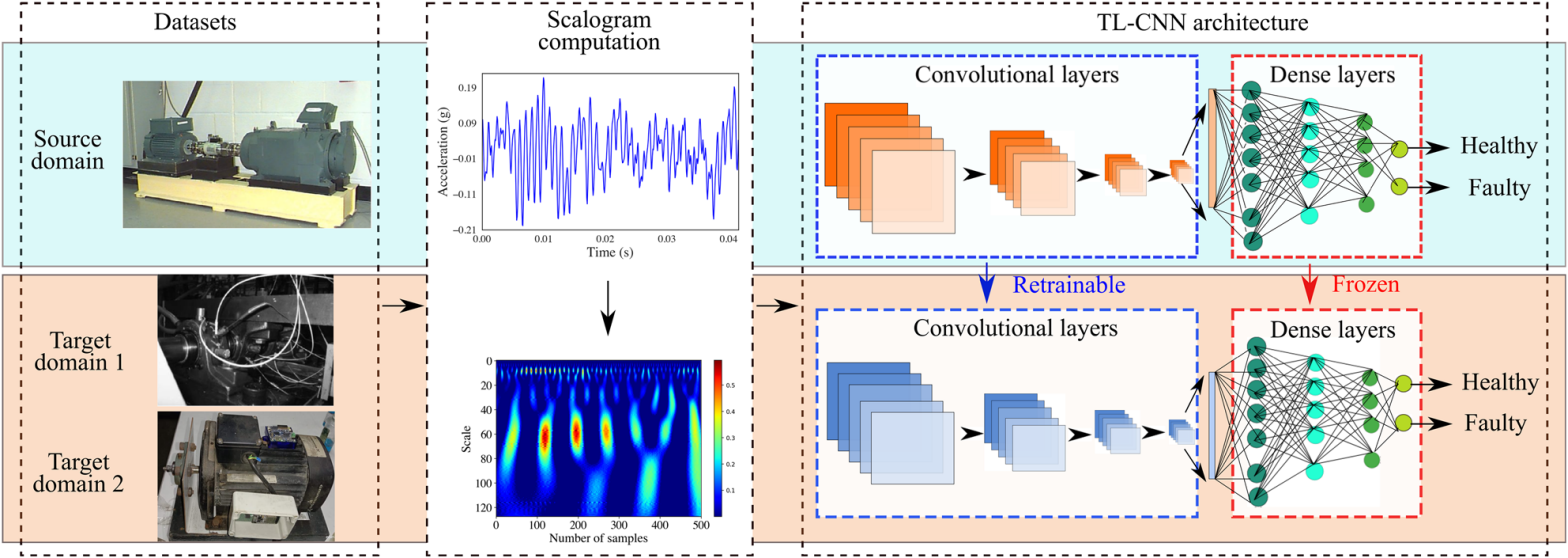
Overview of the TL-CNN architecture presented in this work.

In order to overcome the shortcomings above, in this work, we have implemented a modified TL-based classification model wherein a significantly lesser number of layers are used compared to the conventional pre-trained networks to address the challenges of domain shift and data insufficiency. An overview of the TL-CNN architecture proposed in this work is shown in Fig. 1. The work utilises the widely used Case Western Reserve University bearing dataset (CWRU)^29^ as the source domain dataset for training CNNs on time-frequency domain representations of the vibration signals, namely scalograms. Whereas, two different target domain datasets are considered as the test dataset to demonstrate the versatility of the proposed TL approach: data generated in the lab from a motor instrumented with low precision sensor (LPS), and run-to-failure unlabelled dataset from the Center for Intelligent Maintenance Systems, University of Cincinnati (IMS)^30^. Measurements obtained with low precision sensors along with high variability (greater interquartile range about the mean) make it difficult to distinguish between distributions for healthy and faulty operating conditions. No additional processing of target domain data is required since the source and target data are subjected to the same time-frequency transform. We have evaluated the performance of deep TL models by transferring parameters of convolutional layers and dense layers and studied the sensitivity of retraining of each layer separately. The key contributions of this paper are as follows:

- Our observations indicate that TL using scalograms as input features yields higher accuracy even when the statistical distribution of data obtained with lower precision sensors is not sufficiently distinct for healthy and faulty classes.
- The novelty of the proposed deep TL algorithm is the improvement in prediction ability obtained with a significantly lesser number of trainable parameters (1320) by fine-tuning (retraining) the convolutional layers (i.e. freezing dense layers) to better capture domain-specific abstract information, as compared to the conventional approach of retraining dense layers (i.e. freezing convolutional layers).
- The improvement in accuracy is also observed for unlabelled data with the limited number of samples available for training (IMS).

## Methodology

### Time-frequency based representations of vibration signals

Various methods are used to represent vibration signals in the time and frequency domain. However, time-frequency imaging methods are efficient at capturing the non-stationary nature of the vibration signals^31–34^. In this work, we have considered scalograms obtained using continuous wavelet transform (CWT) as a representation of the vibration signals. CWT provides a more flexible time-frequency resolution as compared to spectrograms obtained with short-time Fourier transform (STFT), by using the mother wavelet function that can be scaled and shifted in correspondence to events in the signals. The scalogram is the absolute value of the CWT of a signal, plotted as a function of scale (*s*) and time translation $(\tau )$:

1 $$\begin{aligned} F(\tau ,s) = \frac{1}{\sqrt{|s|}}\int \limits_{-\infty }^{+\infty } f(t) \psi ^ * \left( \frac{t-\tau }{s}\right) \, dt, \end{aligned}$$

where, $\psi (t)$ denotes the mother wavelet, and the asterisk denotes the complex conjugate. Expanded wavelets (large values of *s*) capture the low-frequency components of the signal with bad time resolution whereas, shrunken wavelets (small values of *s*) capture high-frequency components with good time resolution.

### Description of experimental datasets

Brief descriptions of the source and target domain vibration datasets used in this study are provided below:

(i) *CWRU data set* Among various publicly available machine vibration datasets, CWRU is widely accepted as a standard reference labelled dataset that has been used to validate different fault diagnosis models^29^. Vibration signals from a 2 hp Reliance Electric motor were acquired using high-precision accelerometers with healthy and faulty bearings. Single point faults (inner race, ball bearing, outer race) ranging from 7 to 40 mil in diameter were introduced separately in the test bearings through electro-discharge machining. These faulty bearings were reinstalled into the motor at the drive end and fan end. Sensors were placed at these locations and also at the base end of the motor, and vibration data was recorded at a sampling rate of 12 kHz for motor speeds varying from 1797 to 1720 rpm.
(ii) *IMS data set* The bearing dataset provided by IMS is available for download from the NASA Ames Prognostics Data Repository^30^. The data set comprises of measurements from high-sensitivity quartz ICP® accelerometers, one each installed on four bearings on a shaft. Data from three run-to-failure experiments are provided. The rotation speed was 2000 rpm for a radial load of 6000 lb applied to the shaft. All failures occurred after exceeding 100 million revolutions. Each time series was recorded in an individual file, containing 20,480 data points obtained at 20 kHz sampling rate. Out of the three datasets, we have considered the dataset with the highest number of recordings: channel 3 from test number 3 (culminating in outer race fault). For further details on the experimental setup, please refer report by Qiu et al.^35^. Since the IMS-bearing data set is an unlabelled dataset, the mean peak frequency (MPF) of the spectrogram was used to label the healthy and faulty data, in accordance with the strategy presented by Mukherjee et al.^36^.
(iii) *Lab generated data with low precision sensor (LPS)* This dataset comprises vibration data acquired from a motor running at a speed of approximately 3000 rpm using STMicroelectronics STEVAL-STWINKT1B wireless industrial sensor node that includes an ultra-wide bandwidth (6 kHz), low-noise, 3-axis vibration sensor (STMicroelectronics IIS3DWB) with 26.7 kHz data rate. The dataset contains 650 files with 15,000 samples (i.e. 560 ms duration) each for both the healthy and faulty operating conditions of the motor. The fault was introduced by coupling a bearing with a faulty ball bearing to the motor shaft.

In our study, CWRU was chosen as the source domain dataset, and IMS and LPS datasets were used as the target domain datasets. The CWRU dataset consists of a vast collection of labelled vibration data obtained with high precision sensor corresponding to baseline (healthy) operation, and various types of bearing faults with different fault depths obtained with different motor speeds, and is, therefore, a suitable choice for source domain to incorporate more variation and develop a robust deep CNN based fault classification algorithm. While the IMS dataset is also acquired using a high-precision vibration sensor, it is a run-to-failure dataset consisting of a limited number of vibration recordings in faulty operation (useful for studying the TL model performing with insufficient training data). The LPS dataset is altogether different from the CWRU dataset, consisting of data obtained on a different motor running at higher speed, with a low-cost albeit comparatively lower precision MEMS vibration sensor (useful for performance evaluation of domain adaptation of TL model).

### Transfer learning for machine fault diagnosis

For generating scalograms, the following parameters were used—number of data samples used to generate scalogram: 500, scale factor: 128, wavelet function: Morlet. This resulted in scalogram images of size 128 $\times$ 500. The generated scalograms were normalized using the StandardScaler normalisation method before using them as inputs for the CNN-based classification algorithm. To find the optimum CNN architecture for the training data (CWRU), *RandomSearch* algorithm was employed, wherein the best set of hyperparameters were obtained based on the maximum cross-validation score. Table 1 presents the hyperparameter range used in the *RandomSearch* algorithm and the optimum parameter values obtained from the algorithm that yielded 100% accuracy for fivefold cross-validation with 10 iterations on the training data.

**Table 1:** Hyperparameter tuning of CNN architecture using *RandomSearch* algorithm.

Hyperparameter	Range	Optimal value
No. of conv layers	[2, 3]	2
No. of dense layers	[2, 3]	3
Filters (1${\textrm{st}}$ Conv layer)	[32, 16, 8]	16
Filters (2${\textrm{nd}}$ Conv layer)	[32, 16, 8]	8
Filters (1${\textrm{st}}$ Dense layer)	[32, 16, 8]	16
Filters (2${\textrm{nd}}$ Dense layer)	[32, 16, 8]	8
Learning rate	[0.001, 0.01, 0.1]	0.01
Batch size	[64, 128, 256]	64
No. of epochs	[10, 20, 30]	30

**Table 2:** Summary of CNN architecture for scalograms.

Layer name	Layer (kernel size $\times$ no. of filters)	Output shape	Trainable parameters
L1	Conv (3 $\times$ 3 $\times$ 16)	128 $\times$ 500 $\times$ 16	160
L2	Maxpool (2 $\times$ 2)	64 $\times$ 250 $\times$ 16	0
L3	Conv (3 $\times$ 3 $\times$ 8)	64 $\times$ 250 $\times$ 8	1160
L4	Maxpool (2 $\times$ 2)	32 $\times$ 125 $\times$ 8	0
L5	Flatten	32,000	0
L6	Dropout (0.2)	32,000	0
L7	Dense (16)	16	512,016
L8	Dense (8)	8	136
L9	Dense (1)	1	9
Total trainable parameters			513,481

A detailed summary of the CNN architecture realised utilising the tuned hyperparameters is presented in Table 2. The CNN architecture comprises two convolutional layers and three dense layers. Max-pooling layers were used to reduce feature dimensions while retaining salient information of the inputs in the convolutional layers. The convolutional layers incorporated rectified linear unit (ReLU) activation functions to introduce non-linearity, and zero padding was performed to ensure the same input and output image dimensions. The dropout rate of 0.2 was used to avoid over-fitting to the data. The CNN architecture was trained using Adam optimizer with learning rate $=0.01$ for 30 epochs using binary cross-entropy as the loss function. We observed that the training accuracy and loss converged within 30 epochs. The predicted probabilities were assessed using a sigmoid function to determine the class to which the input data belongs.

Parameter-based TL allows the reuse of parameter weights to improve the accuracy when data distribution differs from the source domain to the target domain. Lower (convolutional) layers of CNNs capture more domain-specific information concealed within the image by convolving it with a kernel (or filter), while deeper (dense) layers are responsible for learning information that is relevant for making the decision^37, 38^. In the TL framework presented in this work, the source classifier CNN was trained on the CWRU dataset, and the target classifier (TL-CNN) was then allowed to leverage this learned information of decision-making by transferring weights of certain layers in the CNN, and retraining the remaining layers with data from the target domain. We explored transferring weights of individual convolutional and dense layers, to study the sensitivity of each layer to the model performance on the target domain. Separately, we also considered TL-CNNs with weights transferred for the dense layers (i.e. all dense layer weights frozen, and convolutional layers re-trained). We considered two different target domain cases for validation of the proposed deep TL architecture i.e., when the target domain dataset is derived from a different machine with low precision sensor (LPS) and when the target domain contains limited and unlabelled data (IMS).

## Results and discussion

**Figure 2:**
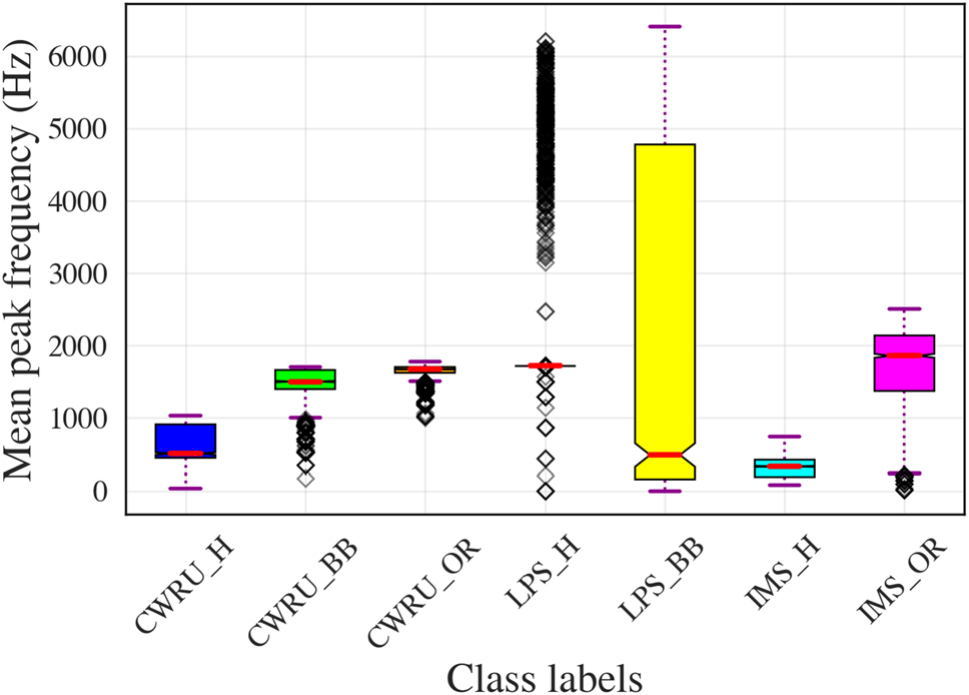
Mean peak frequency (MPF) for vibration data in the three data sets (CWRU, IMS, LPS) corresponding to various operating conditions (*H* healthy, *BB* ball bearing fault, *OR* outer race fault).

Since IMS data contains an outer race (OR) fault and the LPS dataset contains a ball bearing (BB) fault, both these fault conditions were chosen from the CWRU dataset for training CNNs for binary classification of faults. For evaluating the CNNs, we generated 5000 scalograms from each dataset (CWRU, IMS, LPS): 2500 each for healthy and faulty classes. We have used the hold-out cross-validation technique to split the 5000 scalograms from each dataset in the ratio of 50%:20%:30% for training, validation and testing of the CNNs, respectively. Table 3 shows a summary of the results thus obtained. The first four cases in Table 3 i.e., cases (a)–(d), show the model performance when training and test data belong to the same distribution (i.e., same data set). As expected, the accuracy of the CNN models for CWRU and IMS datasets was greater than 99%, owing to the superior performance of CNNs for binary classification of faults in CBM. However, classification accuracy for the LPS dataset i.e., case (d), was lower than CWRU and IMS datasets, which comprise data collected with high-precision sensors. Apart from sensor precision, another factor that contributes to the low classification accuracy in case (d) is the significant overlap in mean peak frequency (MPF) for healthy (H) and ball bearing fault (BB), as seen in Fig. 2. MPF is a key time-frequency domain feature that increases sharply with advancing defects in machines^36^. The MPF for healthy and faulty classes is well-separated in CWRU and IMS datasets. Therefore, it is not surprising that the accuracy and F1 score is lower for case (d) as compared to cases (a)–(c) in Table 3.

**Table 3:** CNN performance results on various data sets using scalograms as inputs.

Case	Train data	Test data	Trainable parameters	Accuracy (%)	F1 score (%)
Training and testing on same data set					
(a)	CWRU (H,BB)	CWRU (H,BB)	513,481	100	100
(b)	CWRU (H,OR)	CWRU (H,OR)	513,481	100	100
(c)	IMS (H,OR)	IMS (H,OR)	513,481	99.6	99.6
(d)	LPS (H,BB)	LPS (H,BB)	513,481	89.73	88.04
Training and testing on different data sets without TL					
(e)	CWRU (H,OR)	IMS (H,OR)	513,481	50	0
(f)	CWRU (H,BB)	LPS (H,BB)	513,481	50	0
Training and testing on different data sets with TL (TL-CNN)					
(g)	CWRU (H,OR)	IMS (H,OR)	1320	98	98
(h)	CWRU (H,BB)	LPS (H,BB)	1320	96.6	96.6

### Impact of retraining individual TL-CNN layers on accuracy

**Figure 3:**
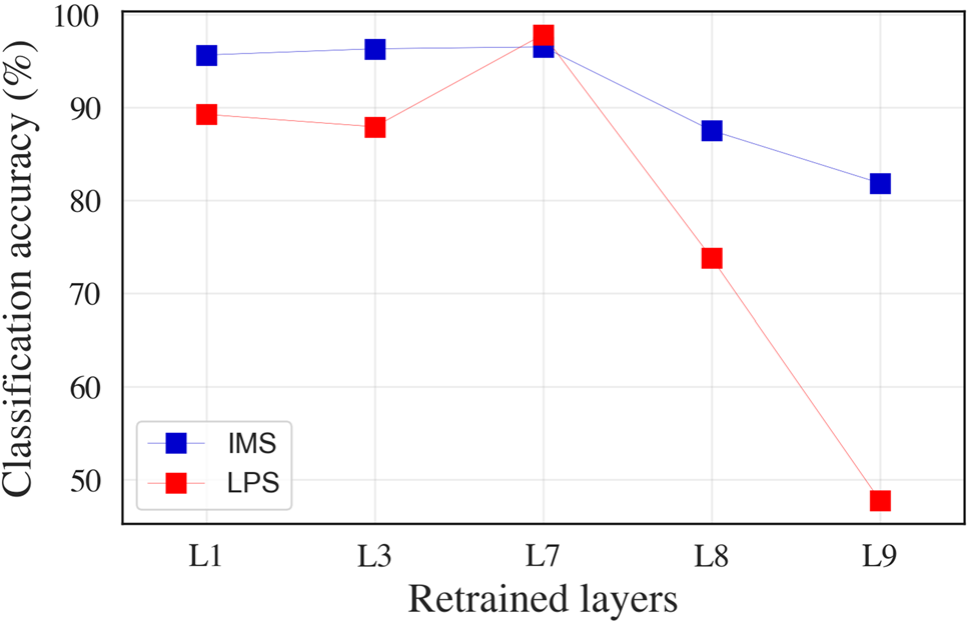
Impact of retraining individual layers in deep TL-CNNs for parameter transfer (while keeping weights of other layers frozen) for scalograms (layer names as per Table 2) used as inputs.

For parameter-based TL, the appropriate transfer of weights from the source to the target domain is critical for improving model performance. The lower (convolutional) layers in CNNs capture domain-specific information, and the deeper (dense) layers contribute to the effective learning required for data classification^37, 38^. Since the source and target domain datasets vary concerning the environment and load conditions, we hypothesise that the CNN architecture can be adapted to these changes by retraining the weights in only the lower layers, while weights in the deeper layers weights can be transferred unmodified for effective fault classification. To validate this hypothesis, we examined the sensitivity of retraining individual convolutional and dense layers in the CNNs, while keeping the weights of other layers frozen (parameter transfer). The impact of retraining weights of individual layers for IMS and LPS target domain datasets is shown in Fig. 3. We observed that the classification accuracy obtained by retraining lower layers was greater than the conventional method of retraining deeper layers in the model. Along with the improvement in accuracy compared to the conventional approach, the number of retrainable parameters is also significantly smaller, which can certainly contribute to improving the training time for the model.

### Performance evaluation of TL-CNN for domain generalisation

The domain generalisation ability of the CNNs trained on the CWRU dataset was abysmal in absence of transfer learning, resulting in poor accuracy and F1 score: cases (e) and (f) in Table 3. Utilising TL-CNN with weights retrained for convolutional layers L1 and L3 (and frozen for dense layers L7, L8, and L9) resulted in significant improvement in domain adaptability, and high accuracy and F1 score for cases (g) and (h). For instance, the accuracy and F1 score improved from 50% and 0% (i.e. case (e) in Table 3) to 98% and 98% (i.e. case (g) in Table 3), respectively; and from 50% and 0% (i.e. case (f) in Table 3) to 96.6% and 96.6% (i.e. case (h) in Table 3), respectively. The number of trainable parameters for cases (g) and (h) were mere 1320, corresponding to the sum total of the number of trainable parameters in convolutional layers in Table 2.

### Impact of the size of target domain data set

**Figure 4:**
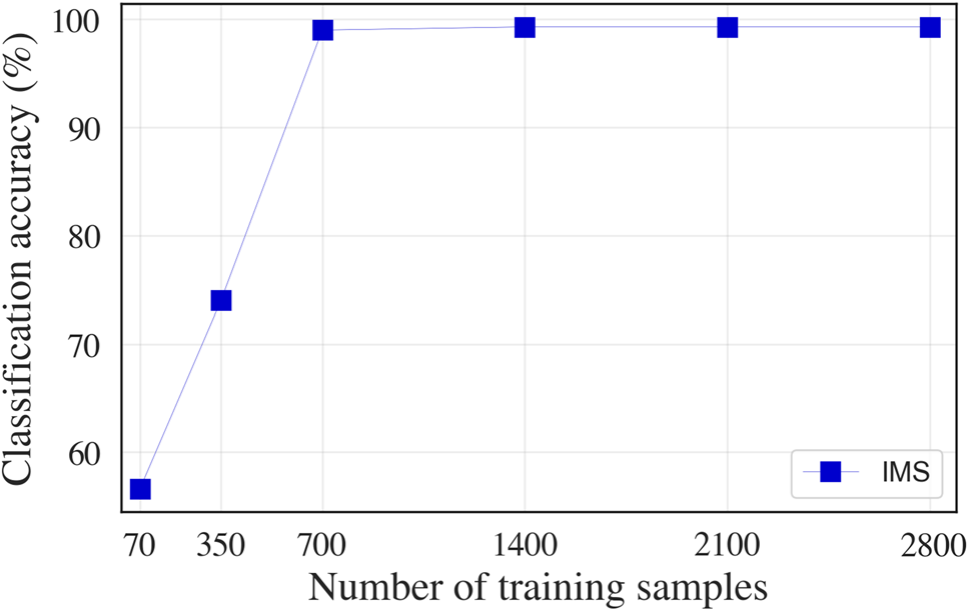
Classification accuracy of TL-CNNs (trained on CWRU, followed by re-training and testing on IMS) for the various number of training samples from the target domain used for retraining the convolutional layers.

As previously mentioned, practical installations of vibration CBM systems result in the generation of a disproportionate amount of healthy data as compared to data in faulty operating conditions due to the abrupt and sudden failure of machines, unlike lab-generated datasets with artificially engineered and controllably introduced faults. Thus, the TL-CNNs may have to be retrained with a small number of samples from healthy and faulty operating conditions. To emulate this scenario, we utilised the TL model performance on the run-to-failure IMS dataset that possesses a large number of healthy samples as compared to faulty samples and used MPF as the signature for assigning labels, using the method described by Mukherjee et al.^36^. Figure 4 presents the accuracy of the deep TL-CNN for a varying number of samples used for retraining the convolutional layers. When the model was retrained using 2800 samples (i.e. scalograms), the accuracy obtained was greater than 95%. When half the number of samples were used for retraining (i.e. 1400), the accuracy was still in excess of 95%, thus highlighting the utility of the TL-CNN for target datasets of limited size and significantly different distribution from the source domain dataset. Note that the TL-CNN showed accuracy in excess of 95% even with as low as 700 samples used for retraining, while the performance of the TL-CNN with scalograms degraded significantly below 700 samples.

**Table 4:** Comparison of the proposed work with other parameter-based transfer learning methods used for domain generalisation for efficient machine fault diagnosis.

References	Method	Input features	Dataset	Application scenarios	Performance remarks
He et al.^39^	Improved deep transfer autoencoder	Raw time-series vibration data	Gearbox fault dataset	Same source and target domain datasets but with varying operating condition	90% accuracy when target domain has significant changes in operating conditions
Lu et al.^40^	AlexNet	Spectrograms	Bearing dataset (CWRU)	Source domain: non-manufacturing data (ImageNet), target domain: manufacturing (bearing data)	99.7% accuracy when target domain has significant changes in operating conditions
Wang et al.^41^	VGG19	Scalograms	Bearing dataset (CWRU)	Source domain: non-manufacturing data (ImageNet), target domain: manufacturing (bearing data)	93% accuracy when target domain comprises different fault types and severities
Li et al.^42^	CNN	Raw time-series vibration data	Bearing dataset (CWRU), gearbox fault dataset	Source and target domain datasets with variour operating conditions and distinct fault components	$>90$% accuracy when all CNN layers are fine-tuned with target domain data (lower accuracy when only last layer is fine-tuned)
Chen et al.^43^	CNN	Raw time-series vibration data	Bearing dataset (CWRU), gearbox fault dataset, and lab generated bearing data	Source and target domain datasets across different machines and varying operating conditions	99% accuracy with all training samples (accuracy number drops with reduced number of training samples)
This work	Light-weight CNN	Scalograms	Bearing dataset (CWRU), run-to-failure bearing data (IMS), lab generated data with low precision sensor (LPS)	Source and target domain datasets across different machines and sensors of different precision	98% accuracy for high precision, unlabelled sensor data in target domain and 96.6% accuracy with labelled, low precision sensor data in target domain, $>95$% accuracy even with halving number of samples used for retraining

Table 4 compares various recent parameter-based TL techniques used for domain generalisation for efficient machine fault diagnosis. He et al. have proposed an improved deep transfer autoencoder-based TL method to enhance classification accuracy in diagnosing gearbox faults even in the presence of significant change in the working condition but when the source and target domain data belong to the same dataset^39^. Lu et al. have used an AlexNet-based pre-trained network to automatically identify and classify distinct bearing faults with improved accuracy for varying load conditions utilising the time-frequency features (spectrograms) generated from the raw time-series vibration signals^40^. Yet in another study, a pre-trained VGG19 network has shown adequate prediction to diagnose different types of bearing faults even when the network is trained on the non-manufacturing specific data (ImageNet dataset)^41^. However, as the source domain data used for training the network are significantly different from the target domain vibration encodings of the machines (spectrograms and scalograms), a large number of trainable parameters are required for domain adaptation thus, increasing computation cost. A joint diagnostic model comprising a CNN wherein the knowledge learned on one fault (bearing) is applied to efficiently predict another fault (gearbox) has been successfully demonstrated by Li et al.^42^. Chen et al. have presented a CNN-based TL model utilising raw vibration data that has shown improved domain generalisation capability when the source and target domain dataset belongs to different machinery and even with varying working conditions^43^. Although a significant improvement in domain generalisation is achieved using the parameter-based TL techniques, in most of the reports, a publicly available vibration dataset acquired using high-precision sensors is used to validate the effectiveness of the models which limits the deployment of most of the models in a real-world scenario of limited access to resource utilisation.

## Conclusion and future work

In summary, in the proposed work we have presented a deep TL-based model for efficient domain generalisation of CNNs trained on a widely accepted benchmark-bearing fault dataset, for high classification accuracy when tested to different distribution target domain vibration CBM datasets obtained from different machines and operating conditions. We studied the performance of the model using a scalogram (time-frequency representation of vibration signal) as input due to its flexible time-frequency resolution, which offers superior classification results. Besides, the TL-CNNs for scalogram presented in this work utilize only 1320 trainable parameters and are thus suitable candidates for edge-implementation and re-training of these models for autonomous inference at the sensor node e.g. Raspberry Pi single board computer. Additionally, we have demonstrated that deep TL-CNNs can be helpful to improve the accuracy of fault classification for unlabelled datasets with few samples available for training. In future work, we aim to explore methods for data fusion from multiple source domains to enable learning of diverse operating conditions to improve domain generalisation further and mitigate *negative transfer*, a common drawback of TL models, wherein the performance of the model on older data is compromised when retrained on newer data that may have different distribution due to concept drift^44^. The proposed technique of transfer learning allows attaining efficient generalisation capability for distinctly distributed target domain datasets with a lesser number of trainable parameters and even with a lesser number of training samples albeit the training on the source domain should include the fault class of the target domain dataset for effective supervised fault classification. While the time-frequency representations explored in this work yield high classification accuracy with self-learned features, they require computing resources for inference, that may be beyond the reach of low-cost edge devices such as microcontrollers. We shall also explore feature engineering strategies to capture the variance in such diverse data sets for developing edge-based compact models^45^, which could truly make a huge impact on vibration CBM. Along with that, we also aim to study and incorporate explainability into such machine learning models, for providing automated and actionable feedback with limited intervention from domain experts.

## Data Availability

The CWRU and IMS datasets analysed in the current study are available at https://engineering.case.edu/bearingdatacenter and https://www.nasa.gov/content/prognostics-center-of-excellence-data-set-repository, respectively. The LPS dataset analysed in the current study is available from the corresponding author upon reasonable request.
